# Historical Sustenance Style and Social Orientations in China: Chinese Mongolians Are More Independent Than Han Chinese

**DOI:** 10.3389/fpsyg.2020.00864

**Published:** 2020-05-08

**Authors:** Ivana Stojcic, Qingwang Wei, Xiaopeng Ren

**Affiliations:** ^1^Key Laboratory of Behavioral Science, Institute of Psychology, Chinese Academy of Sciences, Beijing, China; ^2^Department of Psychology, University of Chinese Academy of Sciences, Beijing, China; ^3^The Department of Psychology, Renmin University of China, Beijing, China; ^4^The Laboratory of The Department of Psychology, Renmin University of China, Beijing, China

**Keywords:** Han Chinese, Mongolian, ecology, interdependence, independence

## Abstract

In this study, we examined the Chinese Han and Mongolian, two ethnic groups that belong to the same national and geographic regions but vary in their degrees of social interdependence and independence. We assumed that the Mongolian, who have traditionally been known as a herding community, exhibit a greater independent social orientation compared to the Han Chinese, who have traditionally been known as an agrarian community. Through three different studies we used the explicit measurement of self-construal, implicit cultural task of self – inflation and the practice of name-giving (i.e., baby names as a cultural product) to test our hypothesis. The obtained results revealed that compared to Han Chinese, Mongolian scored higher on independent – self subscale, had greater levels of self-symbolic inflation and were less likely to give common names to their babies. These findings suggest that Mongolians are more independent than Han Chinese. In view of that, the present study contributes to a better understanding of the complexity of Chinese culture in terms of interdependence and independence, and provides further support for historical sustenance theory.

## Introduction

**FIGURE 1:**
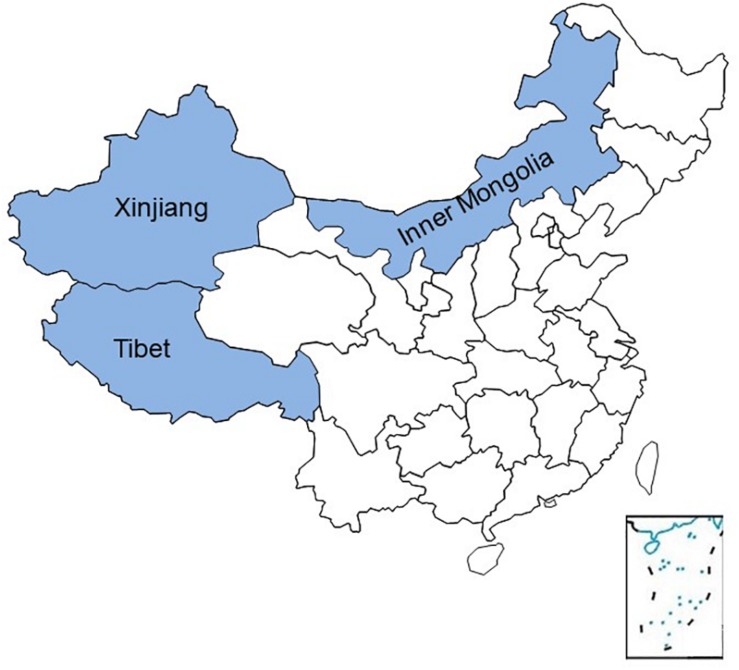
Map of China showing the exact location of regions of Inner Mongolia, Tibet, and Xinjiang, which accommodate the majority of pastoral communities in China.

In a recent study on the differences between individualism and collectivism in China, Talhelm et al. (2014) argued that the differences they observed between the independent northern Han Chinese and the interdependent southern Han Chinese are mainly caused by wheat vs. rice farming, which have historically been dominant economic activities in these two parts of China. While Talhelm’s study provided a great contribution on within-country differences in China, this theory did not include a vast number of Chinese populations and territories. Besides the Han Chinese, China is home to 55 other ethnic minority groups (Clothey, 2005), and farming is not practiced in a substantial portion of the Chinese territory. For example, China has some 260 pastoral counties that accommodate about 39 million people (Miller, 2002), most of which are situated in the regions of Inner Mongolia, Tibet, and Xinjiang and have been excluded from the above-mentioned study (Figure 1). The existing literature argues that herding activities promote independence and analytical cognitive styles (Uskul et al., 2008); nonetheless, the empirical evidence these studies report applies to current ecological systems. The aim of the present study was to learn what happens to cultures after they no longer actively engage in the traditional ecological activities. We were particularly interested in the Chinese setting, especially in view of the newly proposed rice theory. Accordingly, we compared the largest ethnic group in China, the Han Chinese, with a traditionally herding minority ethnic group, the Mongolian, to contribute to the knowledge of social cognition across China and to further validate the relevance of the historical subsistence theory in the field of psychology.

### Individualism and Collectivism

Individualism and collectivism are unquestionably the most common theoretical and empirical concepts in current cross-cultural psychology research. Thus far, they have been used to interpret, clarify, and predict variances in attitudes, behaviors, morals, perception, reasoning, communication, attribution, social interactions, and self-construal (Kagitçibasi, 1997; Oyserman et al., 2002; Green et al., 2005; Heine et al., 2008). Furthermore, these are multifaceted concepts that have been defined in many different ways. The concept of individualism is usually associated with self-sufficiency, self-reliance, uniqueness, and competition. On the contrary, collectivism is associated with in-group dynamics, a need for social harmony, and conformity with group norms (Hofstede, 1980; Bellah et al., 1985; Markus and Kitayama, 1991; Kim, 1994; Triandis, 1996; Kagitçibasi, 1997). Furthermore, research on individualism and collectivism frequently differentiates cultural and national groups from each other (Hofstede, 1980; Hui and Triandis, 1986; Markus and Kitayama, 1991; Fiske et al., 1998). Individualist cultures promote the importance of being independent, distinctive, influencing others and their environments, remaining free from constraints, and being equal. In contrast, collectivist cultures promote the ideas of affiliation, similarity to others, adjusting to others’ situations, and staying rooted in traditions and obligations (Hofstede, 1980; Bellah et al., 1985; Markus and Kitayama, 1991; Kim, 1994; Triandis, 1996; Kagitçibasi, 1997; Oyserman et al., 2002). In relation to this, the constructs of interdependence and independence are thought to represent the core features of collectivism and individualism, given the centrality of the self for human agency (Fischer, 2009).

### Social Interdependence and Independence

Thus far, the existing literature on collectivism/individualism and independence/interdependence has mainly focused on differences between Western or European Americans and Eastern or East Asian cultures (Triandis, 2001; Kitayama and Uskul, 2011). For instance, European Americans are more likely to successfully handle analytical tasks that require focusing on key targets or multiple targets while ignoring their surroundings (Chua et al., 2005; Savani and Markus, 2012). Alternatively, Eastern Asians are more successful on holistic tasks that require the integration of key targets with background information (Savani and Markus, 2012). Similarly, in Eastern cultures, individuals have shown a tendency to focus more on contextual factors than on individual traits, and they are less likely to assign behaviors to the personal characteristics of an individual compared to Westerners (Morris and Peng, 1994). Furthermore, Eastern Asians have shown to be less likely to minimize the importance of domains in which they did not perform well because contrary to their Western counterparts, poor performance does not tend to affect their self-perception (Heine et al., 1999). According to different researchers, these culturally differing modes of thought and behavior are the result of the extent to which a culture’s social practices emphasize independence or interdependence (Fiske et al., 1998; Kitayama and Duffy, 2004; Oyserman and Uskul, 2008). As previously mentioned, these differences are usually observed in terms of Eastern and Western cultures, somewhat disregarding the potential importance of within-country variations. According to Uskul et al. (2008), exploring variations in cognitive tendencies within an environment with shared identity, ethnicity, or language in relation to variations in social orientations is extremely useful; it allows for enhanced control over a potentially large number of confounding variables (Uskul et al., 2008). This premise has been confirmed by several studies that reported differences between the Uyghur Chinese and Han Chinese in terms of social orientation (Ren et al., 2014), as well as the Hokkaido Japanese and mainland Japanese, where the former were more independent and showed more dispositional bias in attribution (Kitayama et al., 2006). Furthermore, Northern Italians have been shown to be more independent than Southern Italians (Martella and Maass, 2000), and neighboring villages in Turkey were observed to diverge in terms of individual/interdependent orientations based on their main economic activities (Uskul et al., 2008). Finally, northern and southern Chinese appear to diverge in terms of individual/interdependent orientations in view of wheat vs. rice farming (Talhelm et al., 2014).

### Mongolian and Han Chinese: Herders and Farmers

China has been traditionally regarded as a collectivistic country, and the Chinese are seen as more collectivist than most other populations on earth, which is traced back predominantly to their Confucian heritage (Nisbett and Masuda, 2003). Nevertheless, recent research has shown variations in the social orientations of the Uyghur and Han Chinese (Ren et al., 2014) and between the northern and southern Chinese (Talhelm et al., 2014), and Confucianism falls short in explaining these differences (Van de Vliert et al., 2013). The Mongolian have traditionally been known as nomadic herders (Li and Huntsinger, 2011; Wang et al., 2013), while the Han Chinese have been known as a traditional agrarian community (Talhelm et al., 2014). Existing literature has shown that farming communities are more interdependent than less sedentary hunter-gatherer communities, which are more independent (Witkin and Berry, 1975; Uskul et al., 2008). According to subsistence theory, some forms of subsistence-like farming require more functional interdependence than herding. Accordingly, independence and mobility, which are main features of herding activity, make herding cultures more individualistic, while stability and high labor demands make farming cultures more collectivistic (Nisbett et al., 2001). For example, according to Nisbett et al. (2001), China’s fertile plains have traditionally promoted agricultural livelihood, which has been linked with certain social pressures and obligations that have influenced cognitive processes in these communities. These communities place emphasis on family as a primal social unit, as well as one’s social environment, as opposed to an individual’s inner potential and self-realization. In these kind of communities, residential mobility was also very rarely practiced, which furthermore promoted interdependence and a holistic cognitive style (Uskul et al., 2008; Kito et al., 2017).

Following social, economic, and technological changes, traditional economies have made a gradual shift to market, command, or mixed economies worldwide (Mamedov et al., 2016; Amadeo, 2018). Similar thing occurred in Inner Mongolia, where the traditionally nomadic Mongolians have settled in permanent homes as their pastoral economy was collectivized during the Mao Era and some have taken jobs in cities as migrant laborers (Bulag, 2004). According to recently proposed rice theory, which is the extension of subsistence style theory, some forms of subsistence like farming entail more functional interdependence compared to other forms such as herding. The peculiarity of the rice theory is that is applies to rice regions, and not just the people farming rice, i.e., the theory advocates that cultures, which farm rice and wheat over thousands of years transmit those same cultures into the modern era, even in areas where most people no longer engage in the traditional subsistence activity but work in modern industry (Alesina et al., 2013; Talhelm et al., 2014).

Accordingly, we hypothesized that the Mongolians would exhibit a more independent social orientation compared to the Han Chinese. We employed three different studies to test our hypothesis. Study 1 used the explicit measures of self-construal, and implicit measures of self-inflation. Study 2 was replication of Study 1, which was conducted to overcome subsequently identified limitations in Study 1. In Study 3, we investigated the practices of name-giving, i.e., baby names as a cultural product.

*Hypothesis*: Chinese Mongolians will exhibit more independent social orientation compared to Han Chinese.

## Study 1

Study 1 was designed to examine independence and interdependence among the Chinese Han and Mongolian via a self-construal scale (SCS) and a symbolic self-inflation measurement. Cultural task analysis was proposed by Kitayama and Imada (2008) as a useful framework for “understanding how the cultural mandates such as independence and interdependence might influence and eventually shape various aspects of psychological processes.” Cultural tasks, in turn, can be defined as culturally scripted ways of achieving the culture’s mandate (Vallacher et al., 1987; Kitayama et al., 2009).

### Participants

In total, 89 Mongolian individuals (20 men and 69 women) and 113 Han Chinese individuals (42 men and 71 women) participated in the study. The participants were recruited via advertisements that were posted on different university campuses in Beijing. The ages of the Mongolian participants ranged from 17 to 28 years (*M* = 21.90, *SD* = 1.94), while for the Han they ranged from 16 to 20 years (*M* = 18.35, *SD* = 0.71). All the scales were administered in Mandarin Chinese, which is the official language of the People’s Republic of China.

### Measures

#### Social Orientation (Interdependence vs. Independence)

The Self-Construal Scale (SCS) (Singelis, 1994) is a well-validated scale used to measure explicit social orientation. It has total of 24 items, which are furthermore divided into two subscales evaluating independent self (e.g., “I enjoy being unique and different from others in many respects”) and interdependent self (e.g., “even when I strongly disagree with group members, I avoid an argument”), with 12 items per subscale. It furthermore uses a 7-point scale where 1 stands for “strongly disagree” and 7 stands for “strongly agree.” Brislin’s (1970) back-translation method was employed for scale translation. The internal consistency of the scale was α = 0.73 for the independent self and 0.84 for the interdependent self, while for the Mongolian it was α = 0.74 for independent self and 0.79 for interdependent self and for the Han Chinese α = 0.71 for independent self and 0.81 for interdependent self.

#### Symbolic Self-Inflation

We used the sociogram task to measure implicit individualism (Kitayama et al., 2009). In this task, participants were asked to make a drawing of their social network. They were asked to use circles to illustrate themselves and their friends; and then use lines to illustrate the relationships between them (Duffy et al., 2008). They had 5 min to finish the drawings. Afterward, the horizontal diameters of the circles were measured. Symbolic self-inflation was evaluated as the difference between the relative size of the circle illustrating self, and the one illustrating the friend. Greater symbolic self-inflation was interpreted as higher tendency toward independent orientations. Prior studies have found that Americans and Chinese people inhabiting wheat farming regions tend to draw larger self-circles than friend circles (Kitayama et al., 2009; Talhelm et al., 2014).

### Results

Preliminary analyses showed that the Chinese Han and Mongolian were matched for gender, and gender had no influence or interaction with the dependent variables *F*(1, 202) = 1.73, n.s. for independent self, *F*(1, 202) = 0.01, n.s. for interdependent self; *F*(1, 202) = 1.15, n.s. for self-inflation, thus we did not discuss gender any further. Chinese Mongolian (*M* = 21.90, *SD* = 1.95) were older than the Han Chinese (*M* = 18.35, *SD* = 0.76), *t*(202) = 17.86, *p* < *0.001*, so we controlled for this in the analysis with age as a covariate.

#### Social Orientation (Interdependence vs. Independence)

Mixed-design analysis with ethnicity as between-subjects factor, scale type as a within-subjects factor, and age as a covariate showed a significant interaction between scale type and ethnicity, i.e., *F*(1, 202) = 7.03, *p* < 0.01, η*_*p*_*^2^ = 0.04. For interdependent self, there was no difference between the Mongolian (*M* = 5.17, *SD* = 0.69) and Han (*M* = 5.23, *SD* = 0.93) Chinese, *t*(202) = –0.51, *p* = 0.61; for independent self, the Mongolian (*M* = 4.92, *SD* = 0.62) scored higher than the Han Chinese (*M* = 4.55, *SD* = 0.74), *t*(202) = 3.83, *p* < *0.001* (see Table 1).

**TABLE 1:** Differences in independence and interdependence among Chinese Han and Mongolian in Study 1.

	Independence	Interdependence
Mongolian	4.92	5.17
Han Chinese	4.55	5.23

*Independence and interdependence are measured using a 7-point scale.*

#### Symbolic Self-Inflation

A significant difference was found in number of friends between the Han (*M* = 9.55, *SD* = 5.47) and Mongolian (*M* = 5.41, *SD* = 3.42), *t*(202) = 6.12, *p* < 0.001, so the number of friends was controlled in the following analysis.

The width of a circle that designated one’s friends was averaged for each participant and then subtracted from the width of a circle that designated one’s self so that the higher number represented a greater symbolic-inflation. A one-way ANCOVA was performed with ethnicity as between-subject factor and number of friends and age as covariates. The results showed a significant main effect of ethnicity, *F*(1, 202) = 16.93, *p* < 0.001, η*_*p*_*^2^ = 0.08. The Chinese Mongolians (*M* = 0.43, *SD* = 0.56) showed a higher symbolic self-inflation than the Han Chinese (*M* = 0.12, *SD* = 0.79) (see Table 2).

**TABLE 2:** Differences in self-inflation among Chinese Han and Mongolian in Study 1.

	Self-inflation
Mongolian	0.43
Han Chinese	0.12

*Self-inflation scores are expressed in cm.*

We further unpackaged symbolic self-inflation in self-circle and friend-circle. A mixed ANCOVA with ethnicity (Chinese Han vs. Chinese Mongolian) as between-subject factor and circle (self vs. friend) as within-subject factor, controlling for age and number of friends showed a significant interaction effect of self/friend and ethnicity, *F*(1, 202) = 14.96, *p* < *0.001*, η*_*p*_*^2^ = 0.07. The Mongolian drew larger self-circles (*M* = 2.33, *SD* = 0.85) than the Han Chinese (*M* = 2.12, *SD* = 0.70), whereas the Mongolian (*M* = 1.90, *SD* = 0.77) reported smaller friend circles than the Han Chinese (*M* = 2.00, *SD* = 0.74). The interaction effect of self/friend and number of friends was also significant, *F*(1, 202) = 2.21, *p* = 0.003, η*_*p*_*^2^ = 0.05.

Then self-circle and friend-circle were analyzed separately. The self-circle was analyzed by UNIANOVA, where ethnicity was entered as between-subject factor, controlling for age and number of friends. The results showed that the main effect of ethnicity was significant, *F*(1, 202) = 6.01, *p* = 0.015, η*_*p*_*^2^ = 0.03; and the main effect of number of friends was not, *F*(1, 202) = 1.04, *p* = 0.31. The friend-circle was analyzed by UNIANOVA, where ethnicity was used as between-subject factor, controlling for age and number of friends. The results showed that the main effect of ethnicity was not significant, *F*(1, 202) = 1.35, *p* = 0.25, while the main effect of number of friends was, *F*(1, 202) = 16.86, *p* < 0.001, η*_*p*_*^2^ = 0.08. This suggested that the number of friends had effect on friend-circle but no effect on self-circle.

### Discussion

The obtained results supported our hypothesis that Chinese Mongolians are more independent than Han Chinese, not only in their self-reported explicit beliefs but also in symbolic self-inflation. These results were consistent with those reported by Uskul et al. (2008), where herders in Turkey appeared more independent than farmers. The obtained results supported the premise that historical subsistence systems could lead to differences in self-construal.

Nonetheless, in Study 1 we failed to control for the exact provenance of the Han Chinese students, which might lead to concluding that the observed differences are related to residential mobility rather than historical subsistence cultural backgrounds. While the Mongolians move to Beijing for educational purposes, so do the Han Chinese from all over China due to the number of prestigious universities there, which might suggest that both the Chinese Han and Mongolian have experienced geographical mobility. Similarly, the observed differences might also be ascribed to north-south differences, as the Mongolians mainly come from north China while the Han Chinese came from all over China. Previous study has suggested that northern Han Chinese are more individualistic than the southern Han Chinese; however, it is very dubious whether these differences would change the obtained results. First, herder/farmer differences are stronger than within-farmer differences in general (Barry et al., 1959; Goldschmidt, 1971; Nisbett et al., 2001; Nisbett and Masuda, 2003; Talhelm and Oishi, 2014). Second, Talhelm found that compared with southern cities like Shanghai, migrants in northern cities, such as Beijing, more frequently come from northern regions (Talhelm and Oishi, 2014). Nevertheless, it was necessary to account for north-south differences to exclude the possibility of geographical effects, which we did in Study 2.

## Study 2

Study 2 was designed to overcome the limitations subsequently detected in Study 1. Just as in Study 1, we used a SCS and a symbolic self-inflation measurement in order to examine independence and interdependence among the Chinese Han and Mongolian controlling for age and the provenance of the participants.

### Participants

In total, 100 Chinese Mongolians (43 men and 56 women) and 136 Han Chinese (49 men and 86 women) participated in the study. The participants were recruited via advertisements that were posted on different university campuses in Inner Mongolia, and all of them were from Inner Mongolia. The ages of the Mongolian participants ranged from 18 to 26 years (*M* = 20.34, *SD* = 1.97), while for the Han Chinese they ranged from 18 to 30 years (*M* = 20.33, *SD* = 2.04). All the scales were administered in Mandarin Chinese, which is the official language of the Republic of China. The measures used in Study 2 are the same as those in Study 1. After answering questions from these scales, participants were asked to answer sociodemographic questions related to the highest level of educational attainment of their parents (primary school; secondary school; college; bachelor degree; master degree or higher), and their own average monthly income in RMB (<1,000; 1,000–2,000; 2,000–3,000; 3,000–4,000; 4,000–5,000; 5,000–10,000; 10,000–20,000; 20,000–50,000; 50,000–100,000; more than 100,000).

### Results

A chi-square test of independence was performed to examine whether there were any gender differences between Chinese Han and Mongolian. The obtained results revealed that gender differences among Chinese Han and Mongolian were not statistically significant, *X*^2^(2, *N* = 235) = 2.57, *p* = 0.276, nor was the age difference *t*(232) = -0.38, *p* = *0.97*. Furthermore, Han Chinese were significantly richer (*M* = 5.66, *SD* = 2.20) compared to Mongolians (*M* = 3.47, *SD* = 1.46); *t*(235) = 8.69, *p* < *0.001.* Han Chinese’s parents also had significantly higher levels of education (*M* = 3.48, *SD* = 1.27) compared to Mongolian (*M* = 2.99, *SD* = 0.95); *t*(235) = 3.38, *p* < *0.001*.

#### Social Orientation (Interdependence vs. Independence)

Mixed-design analysis with ethnicity as between-subjects factor, and scale type as a within-subjects factor showed a significant interaction between scale type and ethnicity, i.e., *F*(1, 227) = 10.95, *p* < 0.001, η*_*p*_*^2^ = 0.04. For interdependent self, there was no difference between the Chinese Mongolian (*M* = 5.26, *SD* = 0.57) and Han (*M* = 5.13, *SD* = 0.66), *t*(234) = –1.51, *p* = 0.13; for independent self, the Chinese Mongolian (*M* = 4.92, *SD* = 0.67) scored higher than Han Chinese (*M* = 4.47, *SD* = 0.64), *t*(234) = –5.25, *p* < *0.001* (see Table 3).

**TABLE 3:** Differences in independence and interdependence among Chinese Han and Mongolian in Study 2.

	Independence	Interdependence
Mongolian	4.92	5.26
Han Chinese	4.47	5.13

*Independence and interdependence is measured using a 7-point scale.*

#### Symbolic Self-Inflation

The width of a circle that designated one’s friends was averaged for each participant and then subtracted from the width of a circle that designated one’s self so that the higher number represented a greater symbolic-inflation. An independent samples *t*-test revealed that Mongolian (*M* = 0.59, *SD* = 0.76) showed higher symbolic self-inflation compared to Han Chinese (*M* = 0.23, *SD* = 0.74); the observed differences were statistically significant *t*(235) = -3.71, *p* < 0.001 (see Table 4).

**TABLE 4:** Differences in self-inflation among Chinese Han and Mongolian in Study 2.

	Self-inflation
Mongolian	0.59
Han Chinese	0.23

*Self-inflation scores are expressed in cm.*

We further unpackaged symbolic self-inflation in self-circle and friend-circle. A mixed ANCOVA with ethnicity (Han vs. Mongolian) as between-subject factor and circle (self vs. friend) as within-subject factor showed a significant interaction effect of self/friend and ethnicity *F*(1, 234) = 13.75, *p* < 0.001, η*_*p*_*^2^ = 0.05. The Mongolian drew larger self-circles (*M* = 2.81, *SD* = 0.85) than the Han Chinese (*M* = 2.56, *SD* = 0.77), whereas the Mongolian (*M* = 2.22, *SD* = 0.61) reported smaller friend circles than the Han Chinese (*M* = 2.33, *SD* = 0.71).

Finally, we performed the analysis of covariance to determine a statistically significant difference between Chinese Han and Mongolian on symbolic self-inflation, whilst controlling for income and participants’ parents education. The obtained results revealed a significant effect of ethnicity on levels of self-inflation *F*(1, 232) = 11.99, *p* < 0.001, even after controlling for the effects of income *F*(1, 232) = 2.05, *p* = 0.153 and parents’ education levels *F*(1, 232) = 0.50, *p* = 0.477, neither of which were significantly related to levels of self-inflation.

### Discussion

The results obtained in Study 2 were consistent with the Study 1, providing further support for our hypothesis that Chinese Mongolian are more independent than Han Chinese in both their self-reported explicit beliefs and symbolic self-inflation.

Moreover, we found differences in family income and parents’ education between two groups. Previous studies have shown that people with high SES tend to be more independent and self-inflated compared to those with low SES (Na et al., 2010; Talhelm et al., 2015). Nonetheless, after controlling for these variables, the differences in independence between Chinese Han and Mongolian remained.

## Study 3

In order to furthermore increase the ecological validity of the current research, in Study 3 we tested our hypothesis in real life setting by investigating the baby naming practices between the Chinese Han and Mongolian. Previous studies have pointed out that baby names are a valuable cultural product, which can successfully reveal societal orientation tendencies, as well as cultural change (Twenge et al., 2010; Varnum and Kitayama, 2011; Ogihara et al., 2015), whilst avoiding self-report biases (Van de Mortel, 2008). Individuals with more independent selves and individualist tendencies are inclined to give more unique and unusual names to their newborn babies, unlike those from more collectivist and interdependent cultures that have the opposite tendency, i.e., prefer more common names (Varnum and Kitayama, 2011). Higher percentages of babies receiving common names correspond to fewer parents giving unusual names, and thus, presumably an emphasis on fitting in; lower percentages of babies with common names correspond to stronger emphasis on standing out. From this perspective, the percentage of most common first name can be identified as a national or cultural indicator of individualistic/collectivistic tendencies. Here we assumed that the percentage of most common first names would be lower in Chinese Mongolian compared to Han Chinese.

### Data Collection

We gathered data on popular baby names from National Citizen Identity Information Center (NCIIC) managed by China’s Ministry of Public Security (MPS). The data were obtained as part of the program designed for issuing smart identity cards in the early 2000s, which replaced the old paper versions. The MPS records and manages the information (including name, address, etc.) of all citizens, so these data are a valid source of names given at birth, and they include the whole Chinese population including Chinese Han and Mongolian, with the latter ones mainly inhabiting the Inner Mongolia Autonomous Region of China.

Using this database, we collected data on the number of live births among the Chinese Han and Mongolian from province of Inner Mongolia. This province has a population of about 24 million people, where 17.1% of people are Chinese Mongolian and 79.5% are Han-Chinese according to the 6th population census. We collected and analyzed the most common Han-Chinese male/female names in a total of 19,141,651 Han Chinese (9,811,188 men and 9,330,463 women) and 4,525,209 Mongolian (2,260,285 men and 2,330,463 women). We then computed the percentages of the three naming variables (babies given most common names, babies given top 10 common names, babies given top 20 common names) for both groups according to gender. Owing to the approach we adopted, it was not necessary to calculate the inter–rater reliability.

### Procedure

Following Twenge’s and Varnum’s method, we calculated the percentage of the most common first names for both groups separately by gender (Twenge et al., 2010; Varnum and Kitayama, 2011) using the following formula:

$$t\,h\,e\,t\,o\,p\,X\,m\,o\,s\,t\,c\,o\,m\,m\,o\,n\,f\,i\,r\,s\,t\,n\,a\,m\,e\,s_{\left(g,e\right)}\;=\frac{X_{\left(g|e\right)}}{p\,o\,p\,u\,l\,a\,t\,i\,o\,n_{\left(g|e\right)}}\times 100\%$$

where *X* equals to the number of most common first names; *G* equals to gender (male or female), and *E* refers to ethnicity (Han-Chinese and Chinese Mongolian). For example, the most common male first name in Han-Chinese was Jun (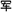); among 9,811,188 Han-Chinese men, 58,652 Han-Chinese were given this name. The percentage of most common first name in Han-Chinese was calculated by dividing the number of people with the most common first names (58,652) by the whole population of male Han-Chinese (9,811,188) and then multiplying it by 100 to obtain the percentage. Finally, we found that the most common name Jun (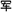) was given to 0.60% of Han-Chinese males.

### Results

The respective percentages were calculated for top 1 and top 10 and top 20 most common first names by gender and by ethnicity, respectively. The relevant data are reported in Tables 5, 6.

**TABLE 5:** Twenty most common first names and their percentage among Chinese Han and Mongolian men.

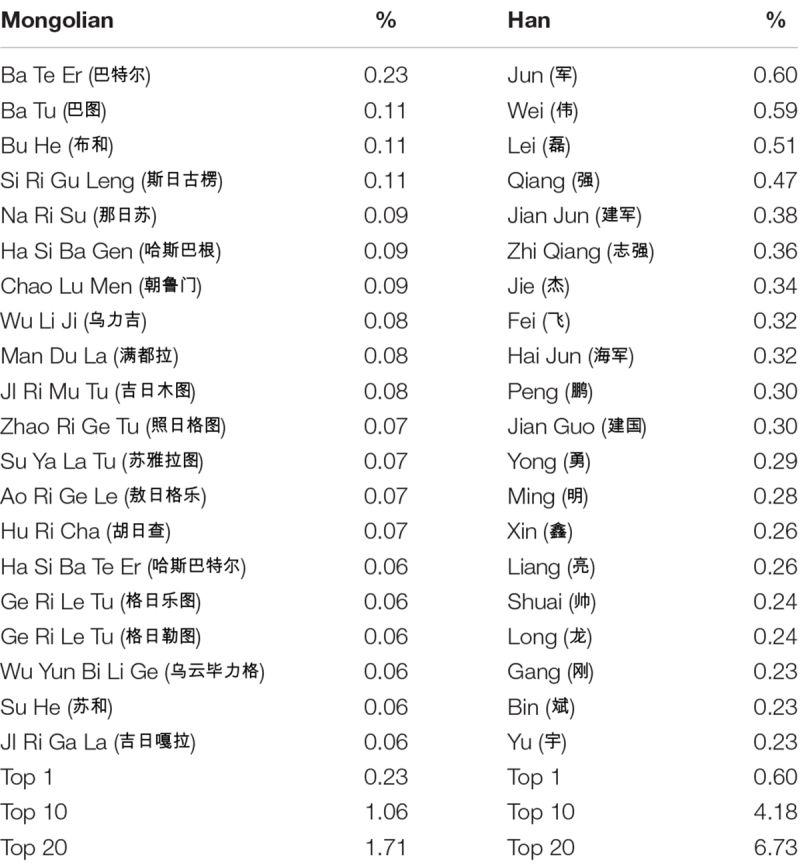

*The names in brackets are written using Chinese characters, top 1, percentage of most common first names; top 10, percentage of 10 most common first names; top 20, percentage of 20 most common first names.*

**TABLE 6:** Twenty most common first names and their percentage among Chinese Han and Mongolian men.

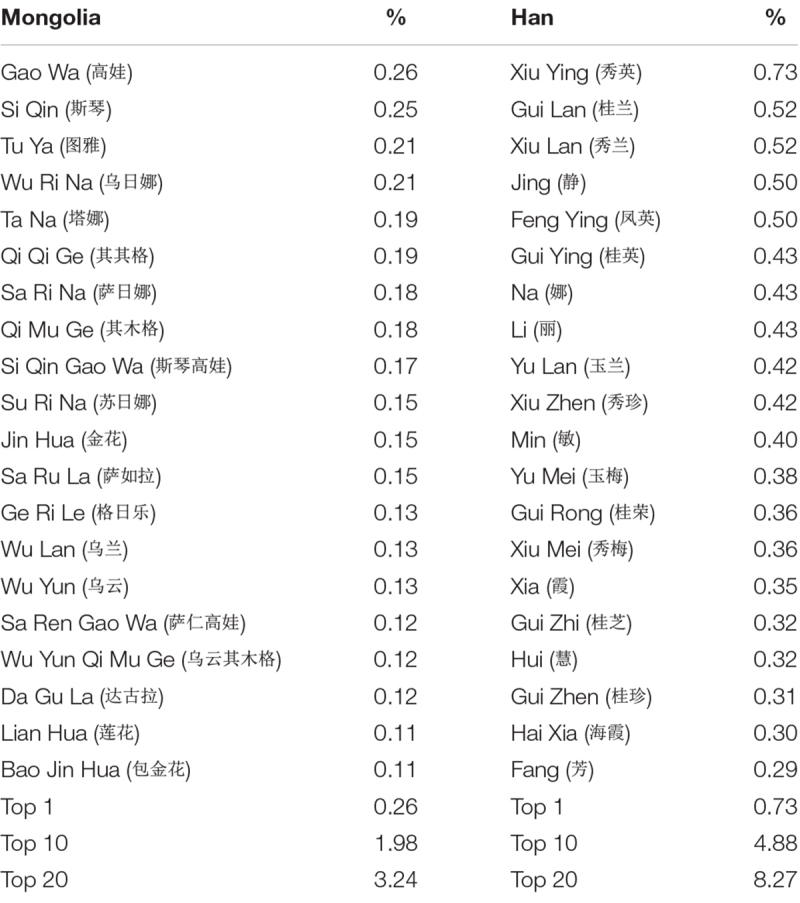

*The names in brackets are written using Chinese characters, top 1, percentage of most common first names; top 10, percentage of 10 most common first names; top20, percentage of 20 most common first names.*

**FIGURE 2:**
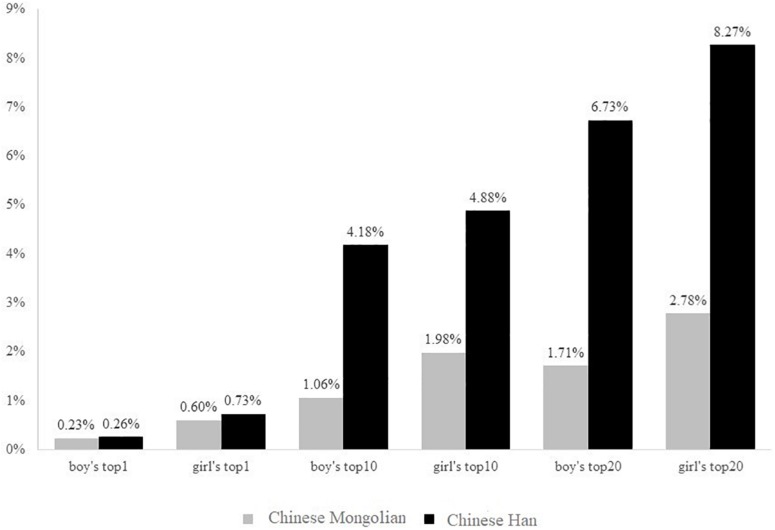
The percentages of the most common name, the most common 10 names, and the most common 20 names among Han Chinese and Mongolian women and men.

A chi-square test of percentages of most common names between Chinese Han and Mongolians was performed to examine the relationship between ethnicity and tendency toward giving more unique names. For the percentage of the most popular male name, *X*^2^(1, *N* = 12,071,473) = 4853.534, *p* < 0.001, Mongolian (0.23%) scored lower than Han Chinese (0.26%). For the percentage of the most popular female name, *X*^2^(1, *N* = 11,595,387) = 6298.957, *p* < 0.001, Mongolian (0.60%) scored lower than Han Chinese (0.73%). For the percentage of 10 most popular male names, *X*^2^(1, *N* = 12,071,473) = 51529.497, *p* < 0.001, Mongolian (1.06%) scored lower than Han Chinese (4.18%). For the percentage of 10 most popular female names, *X*^2^(1, *N* = 11,595,387) = 37122.606, *p* < 0.001, Mongolian (1.98%) scored lower than Han Chinese (4.88%). For the percentage of 20 most popular male names, *X*^2^(1, *N* = 12,071,473) = 84729.228, *p* < 0.001, Mongolian (1.71%) scored lower than Han Chinese (6.73%). For the percentage of 20 most popular female names, *X*^2^(1, *N* = 11,595,387) = 68170.712, *p* < 0.001, Mongolian (2.78%) scored lower than Han Chinese (8.27%) (see Figure 2).

### Discussion

The obtained results revealed that the Mongolian were less likely to give common names to their babies compared to the Han Chinese, thus showing higher levels of independence. The importance of name giving in relation to independent and individualist tendencies has been supported by different studies; e.g., according to a study conducted in USA that examined the cultural change and rise of individualism, common names have become progressively less used from 1950 to 2007 (Twenge et al., 2010); another study conducted in Japan reported a tendency among new parents to pair common Chinese characters with uncommon pronunciations thus creating unique names for babies (Ogihara et al., 2015); according to studies examining frontier settlements and independence, babies were given common names less frequently in western regions of the United States than in its eastern regions. The same pattern was observed in Canada, where common names were less frequently used in western provinces compared to eastern provinces (Varnum and Kitayama, 2011).

In the present study, we included data for Chinese Han and Mongolian living in the same area, i.e., the region of Inner Mongolia, over the span of some 60 years. Since these people have been living in the same region, and thus have shared the same societal circumstances, the most prominent difference between them is ethnicity, which implies settlement of specific areas of Inner Mongolia and traditional engagement in herding or farming activities. Considering that our data cover cultural practices of roughly three generations, that nowadays the traditionally nomadic Mongols have settled in permanent homes as their pastoral economy was collectivized during the Mao Era and that some have taken jobs in cities as migrant laborers (Fairbank and Feuerwerker, 1978; Reardon-Anderson, 2000; Borjigin, 2004), it is logical to assume that the observed differences were the result of the historical subsistence style that was passed on and continued into the modern era (Alesina et al., 2013; Talhelm et al., 2014).

## General Discussion

The purpose of the present study was to contribute to a better understanding of complexity of the Chinese culture in terms of interdependence and independence. We did this by examining social orientations between the Chinese Han and Mongolian. Study 1 and 2 provided support for our hypothesis where the Mongolian appeared more independent than the Han Chinese, not only in self-reported explicit beliefs but also in symbolic self-inflation. Similarly, Study 3 showed that the Mongolian were less likely to give common names to their babies compared to the Han Chinese, thus displaying higher levels of independence.

According to Uskul et al. (2008), different human groups inhabit very different ecological areas that influence economic activities, which consequently lead to different cognitive styles. Ecocultural contexts that support social interdependence have a more holistic cognitive style compared to those that promote social independence. Even though China has been traditionally regarded as collectivistic country (Nisbett and Masuda, 2003), there are certain differences in individualism and collectivism across different regions of China. These differences have been observed in both, Eastern and Western cultures. E.g., in the United States, which is considered as a prototypical individualist culture (Hofstede, 1980; Triandis, 2001), the strongest collectivist tendencies were observed in the Deep South, while individualist tendencies were strongest in the Mountain West and Great Plains (Vandello and Cohen, 1999). In Japan similar tendencies were observed between the residents of Hokkaido, where ethnic Japanese had settled in the late 19th century, and the residents of the main islands of Japan (Kitayama et al., 2006).

### Ecological Systems

Previous studies have identified differences in social orientations between different ethnic groups, i.e., the Uyghur and Han Chinese (Ren et al., 2014) and between Northern and Southern Chinese (Talhelm et al., 2014), which was consistent with our results. We assume the differences observed in the current study are related to the fact that the Mongolian have traditionally been known as nomadic herders (Li and Huntsinger, 2011; Wang et al., 2013), while the Han Chinese have been known as traditional agrarian community (Talhelm et al., 2014). Existing literature has shown that farming communities are more interdependent, compared to less sedentary hunter-gatherer communities that are more independent (Witkin and Berry, 1975; Uskul et al., 2008). According to subsistence theory, some forms of subsistence like farming require more functional interdependence than herding. Accordingly, the independence and mobility which are main features of herding activity make herding cultures more individualistic, while the stability and high labor demands make farming cultures more collectivistic (Nisbett et al., 2001).

In the late1990s and early 2000s, the Chinese government started issuing laws that either banned grazing for several months throughout the year or all together. Furthermore, they wanted to make herding families to raise their animals in stables, or to resettle them in urban areas. Consequently, nowadays only a minority of Mongolians still lead this pastoral lifestyle, while the majority have either switched to agricultural farming or have moved to urban areas (Han, 2011). In view of these facts, the present study provided support for the historical subsistence theory, by adding new evidence to growing literature of the deep roots and persistence of cultural traits in China. The observed results imply the long-run effects of ecological system, which is consistent with previous studies that have shown that cultural differences based on historical subsistence style continue into the modern era, even in areas where most people no longer engage in the traditional subsistence activity but work in modern industry (Alesina et al., 2013).

Among most popular competing theories that explain variations in individualism/collectivism are climate-economic theory (Van de Vliert, 2013), minor/major hypothesis (DuBois, 1969) and modernization theory (Hamamura, 2012), none of which were supported by current research data. Mongolian predominantly live in Inner Mongolia, which is an area with demanding climatic conditions (Kottek et al., 2006). According to existing research on climato-economic imprints on Chinese collectivism and climato-economic theory, Inner Mongolia ranks fourth out of 31 examined regions in collectivism (Van de Vliert et al., 2013). Nevertheless, the Mongolian make only 18% of the total population in this area, while the rest of population is Han Chinese. Previous research has proposed that in-group favoritism may drive outgroup discrimination in harsh climato-economic circumstances, which potentially may have an effect on social orientation of the minority group. Accordingly, the people from marginalized groups would be more likely to emphasize collectivistic over individualistic values (Van de Vliert et al., 2009,2013; Van de Vliert, 2011). However, in this case, if ingroup favoritism promoted outgroup hate, climato-economic circumstances would predict that positive ingroup discrimination and negative outgroup discrimination would be stronger among the Mongolian than among the local Han Chinese given that Han Chinese are majority group (Van de Vliert et al., 2013). Furthermore, according to minority/majority hypothesis, minority groups tend to be more interdependent or collectivistic compared to the majority groups (Freeberg and Stein, 1996; Rhee et al., 1996; Gaines et al., 1997). In the present study, the Mongolian appeared more independent than the Han Chinese, thus failing to provide support for this hypothesis. Finally, modernization theory suggests that the rise of individualism comes about as a consequence of an economic growth (Hamamura, 2012). In fact, there are a number of studies that have reported social class being related to independent/interdependent orientations, where those belonging to middle and upper class tend to be more independent compared to working class (Inglehart and Baker, 2000; Kagitçibasi, 2007; Newson and Richerson, 2009; Varnum et al., 2010). Parental education and income are among the main indicators of one’s social standing or a social class (Duncan and Magnuson, 2003; Crosnoe, 2009; Diemer, 2009), and in the current study Mongolians scored lower on both of these indicators compared to Han Chinese. If modernization theory was good fit for our data, the Mongolians would be more interdependent compared to Han Chinese; nonetheless, we observed an opposite trend. Even after controlling for income and education, Chinese Mongolians still resulted more independent compared to Han Chinese, which is to say that modernization theory had no effect on observed Han – Mongolian differences.

In China, there are some 260 pastoral counties, which accommodate about 39 million people (Miller, 2002), and most of which are situated in the regions of Inner Mongolia, Tibet, and Xinjiang that have not been addressed by previous studies. Results obtained in the current study suggest that historical subsistence theory may explain the observed differences in interdependence/independence in areas with traditional herding subsistence systems thus providing more detailed and complete insight into differences in independence/interdependence across China.

Finally, some researchers have questioned the validity and consistency of measurements used to evaluate differences in independence/interdependence in cultural research, thus indirectly questioning reported findings. For example, SCSs were criticized among others for being instable and challenging to interpret (Levine et al., 2003). In the present study, we used three different measurements, i.e., self-construal, cultural task and cultural product, obtaining consistent results on all of them, thus adding further credibility to reported finings. Taken together, the presented data provide multi-source evidence in support of the hypothesis.

## Limitations

There are some limitations in the present study that need to be pointed out. Even though we expected that the Chinese Mongolians would score higher on independent self and lower on interdependent self-compared to the Han Chinese, this result was only observed with reference to independent self^1^. This could be interpreted as a positive adaptation to the environment observed in both the Chinese Han and Mongolian, with the Mongolians being more independent on some domains. Drawing on a Cultural Task Analysis Framework (Kitayama et al., 2009), it is possible that both the Chinese Han and Mongolian have adopted similar cultural tasks on some domains, such as emphasis on family harmony, which would consequently lead to interdependent attitudes or interdependent cultural mandates. Likewise, in other domains they have adopted different cultural scripts and attitudes that eventually led to higher levels of independence observed in the Mongolians. Future studies should specifically investigate which domains are affected by the herding-agrarian hypothesis.

Next, while we followed the recommendation of Twenge’s and Varnum’s method (Twenge et al., 2010; Varnum and Kitayama, 2011), there’s one difference between our approach and theirs. We used the most common names for the population not at 1 year. Nonetheless, since the age did not differ between two ethnic groups, it is quite probable that the age would not moderate the observed tendencies. Future studies should address this issue to further verify our findings and provide more detailed insight into reported tendencies.

Also, despite the fact historical subsistence theory can explain the differences in independence and interdependence, it is impossible to fully exclude some other variables that could have impacted the social orientations of these ethnic groups. For example, previous studies have shown that religion has direct effects on values (Sasaki and Kim, 2011). Accordingly, different researchers have shown that the protestant religion has led to individualism in western cultures, unlike Catholicism, which has promoted community, interdependence, and collectivism (Oyserman et al., 2002). Also, while the Han Chinese do not have formal religion and are predominantly oriented toward Confucianism (Ren et al., 2014), the Mongolian are known to practice the worship of Heaven (80%), Buddhism (12%), Christianity (2%), and Islam (1%). Thus, future research should examine these practices more in detail, along with their potential impacts on the social orientations of these ethnic groups.

Finally, there are nearly 9 million Mongolians in the world, with ~5.8 million living in China, 2.4 million in the People’s Republic of Mongolia, 0.5 million in Russia, and the remainder in other countries, such as Afghanistan, Pakistan, and Iran (Chen and Ji, 2009; National Bureau of Statistics of China [NBS], 2011). Even though the Inner Mongolia is home to the largest Mongolian population, subsequent studies should address sustenance style and social orientation in Mongolians living within other regions of China as well as outside of China, so as to provide more comprehensive insight into differences in social cognition in these populations.

## Conclusion

The present research is relevant as it is the first to examine the differences between independence and interdependence between the Chinese Han and Mongolian, providing better insight into differences in social cognition across China. We examined the cognitive tendencies in an ethnic minority group that, to the best of our knowledge, has not yet been addressed by previous studies. Furthermore, our findings provide further validation for the historical subsistence theory, which links contemporary psychological differences to ancestral differences in subsistence and societal cohesion.

## Data Availability Statement

The datasets generated for this study are available on request to the corresponding author.

## Ethics Statement

This study was carried out in accordance with the recommendations of the Institutional Review Board of the Institute of Psychology, Chinese Academy of Sciences with written informed consent from all subjects. All subjects gave written informed consent in accordance with the Declaration of Helsinki. The protocol was approved by the Institutional Review Board of the Institute of Psychology, Chinese Academy of Sciences.

## Author Contributions

XR conceived the presented idea and supervised the project. IS developed the theory and wrote the manuscript. XR and QW collected and analyzed the data for Studies 1, 2, and 3. XR conceived and planned the experiments, while XR and QW carried out the experiments. All authors discussed the results and contributed to the final manuscript.

## Conflict of Interest

The authors declare that the research was conducted in the absence of any commercial or financial relationships that could be construed as a potential conflict of interest.

## Footnotes

1 Singelis self-construal scale can be used in two ways; independent self and interdependent self can be used as two different dimensions (Kitayama et al., 2009), or they can be employed as a unidimensional self-construal scale by reverse-scoring the interdependence items (Aaker, 2000). In the present study, an independent sample *T*-Test showed that the Chinese Mongolians (*M* = –0.25, *SD* = 0.77) scored higher than the Han Chinese (*M* = –0.69, *SD* = 1.00), *t*(202) = –3.37, *p* = 0.001 on the unidimensional scale, suggesting that the Chinese Mongolians are more independent than the Han Chinese.
